# End-of-life practices in 11 German intensive care units: Results from the ETHICUS-2 study

**DOI:** 10.1007/s00063-022-00961-1

**Published:** 2022-09-28

**Authors:** C. Denke, U. Jaschinski, R. Riessen, S. Bercker, C. Spies, M. Ragaller, M. Weiss, K. Dey, A. Michalsen, J. Briegel, A. Pohrt, C. L. Sprung, A. Avidan, C. S. Hartog, SepNet Critical Care Trials Group—Ethicus 2 Study Group

**Affiliations:** 1 Department of Anaesthesiology and Operative Intensive Care Medicine, Charité Universitätsmedizin Berlin, Campus Virchow, Berlin, Germany; 2 Department of Anesthesiology and Critical Care, Medicine, University Hospital Augsburg, Augsburg, Germany; 3 Department of Internal Medicine, Medical Intensive Care Unit, Universitätsklinikum Tübingen, Tübingen, Germany; 4 Department of Anaesthesiology and Intensive, Care, University of Leipzig Medical Centre, Leipzig, Germany; 5 Technical University Dresden, Department, of Anesthesiology and Intensive Care Medicine, Universitätsklinikum Carl Gustav Carus, Dresden, Germany; 6 Klinik für Anästhesiologie und Intensivmedizin, Universitätsklinikum Ulm, Ulm, Germany; 7 Department of Anesthesiology and Intensive Care Medicine, Hospital of the Bundeswehr Berlin, Berlin, Germany; 8 Department of Anesthesiology, Critical Care, Emergency, Medicine, and Pain Therapy, Konstanz Hospital, Konstanz, Germany; 9 Klinik für Anästhesiologie, LMU Klinikum München, Munich, Germany; 10 Institute of Biometry and Clinical Epidemiology, Charité Universitätsmedizin Berlin, Campus Charité Mitte, Berlin, Germany; 11 Department of Anesthesiology, Critical Care and Pain Medicine, Hadassah Medical Organization and Faculty of Medicine, Hebrew University of Jerusalem, Jerusalem, Israel; 12 Klinik Bavaria Kreischa, Kreischa, Germany; 13 Department of Anesthesiology and Operative Intensive Care Medicine, Charité Universitätsmedizin Berlin; Campus Charité, Berlin, Germany

**Keywords:** Intensive care units, Withholding life-sustaining treatments, Cardiopulmonary resuscitation, Advance directives, Region, Intensivstationen, Vorenthaltung lebenserhaltender Maßnahmen, Kardiopulmonale Wiederbelebung, Patientenverfügungen, Region

## Abstract

**Background:**

End-of-life care is common in German intensive care units (ICUs) but little is known about daily practice.

**Objectives:**

To study the practice of end-of-life care.

**Methods:**

Prospectively planned, secondary analysis comprising the German subset of the worldwide Ethicus‑2 Study (2015–2016) including consecutive ICU patients with limitation of life-sustaining therapy or who died.

**Results:**

Among 1092 (13.7%) of 7966 patients from 11 multidisciplinary ICUs, 967 (88.6%) had treatment limitations, 92 (8.4%) died with failed CPR, and 33 (3%) with brain death. Among patients with treatment limitations, 22.3% (216/967) patients were discharged alive from the ICU. More patients had treatments withdrawn than withheld (556 [57.5%] vs. 411 [42.5%], *p* < 0.001). Patients with treatment limitations were older (median 73 years [interquartile range (IQR) 61–80] vs. 68 years [IQR 54–77]) and more had mental decision-making capacity (12.9 vs. 0.8%), advance directives (28.6 vs. 11.2%), and information about treatment wishes (82.7 vs 33.3%, all *p* < 0.001). Physicians reported discussing treatment limitations with patients with mental decision-making capacity and families (91.3 and 82.6%, respectively). Patient wishes were unknown in 41.3% of patients. The major reason for decision-making was unresponsiveness to maximal therapy (34.6%).

**Conclusions:**

Treatment limitations are common, based on information about patients’ wishes and discussion between stakeholders, patients and families. However, our findings suggest that treatment preferences of nearly half the patients remain unknown which affects guidance for treatment decisions.

**Supplementary Information:**

The online version of this article (10.1007/s00063-022-00961-1) contains supplementary material, which is available to authorized users.

## Abstract (de)

**Hintergrund:**

Auf der Intensivstation (ITS) wird häufig „end-of-life care“ praktiziert, jedoch ist wenig über die alltägliche Praxis bekannt.

**Ziel der Arbeit:**

Die Erfassung der Praxis der „end-of-life care“.

**Methoden:**

Prospektiv geplante Analyse des Datensatzes der deutschen Zentren, die an der weltweiten Ethicus-2-Studie teilnahmen (2015–2016). Eingeschlossen wurden alle ITS-Patienten mit einer Therapiebegrenzung oder die auf der ITS verstarben.

**Ergebnisse:**

Von 1092 (13,7 %) der 7966 Patienten auf 11 multidisziplinären Intensivstationen galten für 967 (88,6 %) Therapielimitationen, 92 (8,4 %) verstarben unter voller Therapie („failed CPR“, erfolglose kardiopulmonale Wiederbelebung), 33 (3 %) mit Hirntod. Von den Patienten mit Therapielimitationen wurden 22,3 % (216/967) von der ITS entlassen. Therapielimitationen waren häufiger „withdraw“ als „withhold“ Entscheidungen (556 [57,5 %] vs. 411 [42,5 %]; *p* < 0,001). Im Vergleich zu Patienten ohne Therapielimitationen waren die Patienten mit Therapielimitationen älter (median 73 Jahre [Interquartilsabstand (IQR): 61–80] vs. 68 Jahre [IQR: 54–77]), waren öfter entscheidungsfähig (12,9 vs. 0,8 %), hatten öfter Patientenverfügungen (28,6 vs.11,2 %) und es gab häufiger Information über ihre Behandlungspräferenzen (82,7 vs. 33,3 %; alle *p* < 0,001). Ärzte gaben an, dass sie Therapielimitationen mit Patienten und Angehörigen diskutierten (91,3 bzw. 82,6 %). Die Behandlungspräferenzen waren bei 41,3 % der Patienten unbekannt. Der häufigste Grund für Therapielimitationen war das Nichtansprechen auf Maximaltherapie (34,6 %).

**Schlussfolgerung:**

Therapielimitationen sind häufig, basieren auf Informationen über Behandlungspräferenzen und Diskussionen zwischen den Beteiligten, Patienten und Angehörigen. Jedoch zeigen die vorliegenden Ergebnisse, dass bei fast jedem zweiten Patienten die Behandlungswünsche unklar sind und somit ohne Einfluss auf die Therapiezielfindung.

## Introduction

In Germany, the use of intensive care services during terminal hospitalizations has increased steadily in recent years, particularly in the older age groups [10]. The culture of medicine has moved from a more paternalistic model to taking a patient’s autonomy into increasing consideration in order to ensure that patient care is aligned with patient goals [11]. While some consider end-of-life decision-making a growing challenge in German intensive care units (ICUs), others see a positive development towards humanized care.

End-of-life decisions are made when goals of care shift from curative to palliative care because of patient’s treatment preferences or prolonged life-sustaining treatment that is no longer beneficial for the patient. Decision-making regarding end-of-life treatments, however, is complex and requires an active process of deliberation and communication among clinicians, the patient and family members [7]. German intensivists perceive a considerable discrepancy between current end-of-life practice and desired practice [28]. The German Civil Code stipulates that the wishes of patients without decision-making capacity are to be determined hierarchically from (1) a written advance directive, (2) prior verbal statements about the preferred type, duration, and circumstances of the treatment in question, or (3) patient’s general statements and values. However, in practice decision-making is fraught with difficulties due to uncertainty of prognosis and ambiguous patient wishes [20]. The objective of this large multicenter study was to observe and characterize end-of-life practices in multidisciplinary German ICUs in 2015–2016 as a subgroup analysis of a world-wide study [3].

## Methods

### Setting

This is a prospectively planned, secondary analysis of the Ethicus‑2 database specifically describing end-of-life practices in German ICUs. The Ethicus‑2 study was a prospective, observational study of 199 ICUs in 36 countries evaluating consecutive adult ICU patients who died or had a limitation of life-sustaining treatment during a 6-month period [3]. German centers were invited to participate through the German SepNet Critical Care Trials Group, a consortium of over 100 physicians and 50 academic and nonacademic hospitals in Germany. Institutional ethics committee approval, with a waiver of informed consent, was obtained from each participating center. The study was registered in the German Clinical Trials Register (DRKS-ID: DRKS00010044).

### Patients

Consecutive adult patients admitted to participating ICUs who died or had any limitation of life-saving treatments over a 6-month period were recruited in each ICU between September 1, 2015, and September 30, 2016 and were prospectively included. Patients were followed up until discharge from the ICU, death, or 2 months from the first decision to limit life-sustaining therapies.

### Study procedure and data collection

Questionnaires and study material were translated into German. A data study form describing practice and communication of end-of-life decisions was completed for each patient by the senior intensivist in each participating ICU who was responsible for the respective end-of-life decisions. Mutually exclusive end-of-life categories were defined previously [3]: withholding (WH) and withdrawing (WD) treatment, shortening of the dying process (SDP), failed cardiopulmonary resuscitation (CPR), and brain death (BD) (Supplementary Table 1). Other data included patient age, gender, clinical characteristics, type and time of treatment limitations, whether discussed with patients or families, information about patient wishes (meaning any kind of statement about what the patient may want), concurrence with known patient wishes, and reasons as well as obstacles for treatment decisions.

To describe ethical practice, 12 variables were assessed post hoc as described previously [24]. Items represent structured ethical practice, guidelines and legislation (end-of-life practice score [EPS]) ([19]; Supplementary Table 2). Each positive answer received 1 point. The sum was operationalized as an ICU-specific ethical practice score with a range of 0 to 12 points.

### Statistical analysis

Treatment limitations were categorized hierarchically according to the most active limitation (WD > WH). Since there was only one patient in the SDP category, this patient was included in the withdrawing treatment category.

For categorical variables, we report numbers and proportions within end-of-life groups. For continuous variables, we report medians and interquartile ranges. Differences between groups were tested with the Wilcoxon–Mann–Whitney test or the chi^2^ (χ^2^) test. All analyses are performed using the statistical software R [27].

## Results

### Centers

Eleven ICUs participated in this study. Nine were mixed medical/surgical, one was medical, and one was a neurosurgical ICU. Nine centers were in academic hospitals (Appendix).

### Patient population

**Fig. 1:**
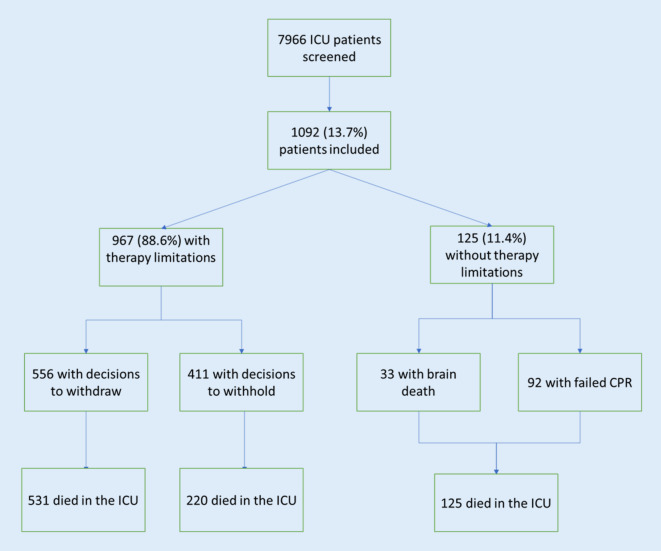
Flow chart. *ICU* Intensive care unit. *CPR* cardiopulmonary resuscitation

Among 7966 consecutively screened patients, 1092 (13.7%) patients were included with treatment limitations (WH or WD), failed CPR or BD (Fig. 1). The median age was 72 (IQR 60–80) years and 647 (59.2%) patients were male. The most common reasons for admission were respiratory (37%) and nontrauma surgical (35.6%). A total of 229 (21%) patients had sepsis on admission and 188 (17.2%) had a diagnosis of cancer. In addition, 126 patients (11.5%) had mental decision-making capacity at the time of decision-making and 270 (26.9%) patients had advance directives (Table 1).

**Table 1:** Study population

Characteristics	All patients	Without treatment limitations	With treatment limitations	*p*
*Total, n (%)*	1092	125 (11.4%)	967 (88.6%)	
*Age in years, (median, IQR)*	72 (60; 80)	68 (54; 77)	73 (61; 80)	< 0.001
*Male, n (%)*	647 (59.2)	76 (60.8)	571 (59)	0.78
*Patient religion*, *n* (%)^a^				
Catholic	132 (12.1)	14 (11.2)	118 (12.2)	
Protestant	54 (4.9)	4 (3.2)	50 (5.2)	
Greek Orthodox	2 (0.2)	0 (0.0)	2 (0.2)	
Islam	19 (1.7)	2 (1.6)	17 (1.8)	
Jewish	1 (0.1)	0 (0.0)	1 (0.1)	
Other	11 (1.0)	2 (1.6)	9 (0.9)	
None	50 (4.6)	5 (4.0)	45 (4.7)	
Unknown	822 (75.3)	97 (77.6)	725 (75.0)	
*Reason for admission*, *n* (%)^b^				
Respiratory	404 (37.0)	31 (24.8)	373 (38.6)	0.004
Surgical (nontrauma)	389 (36)	45 (36)	344 (35.6)	1
Neurological	258 (23.6)	23 (18.4)	235 (24.3)	0.177
Cardiovascular	229 (21.0)	41 (32.8)	188 (19.4)	< 0.001
Medical other than above	498 (45.6)	50 (40.0)	448 (46.3)	0.214
Sepsis at admission	229 (21.0)	19 (15.2)	210 (21.7)	0.117
*Comorbidities*, *n* (%)^b^				
Cardiovascular	729 (66.8)	75 (60)	654 (67.6)	0.109
Respiratory	268 (24.5)	26 (20.8)	242 (25)	0.353
Neurological	211 (19.3)	11 (8.8)	200 (20.7)	0.002
Kidney and urinary tract	205 (18.8)	14 (11.2)	191 (19.8)	0.029
Cancer	188 (17.2)	16 (12.8)	172 (17.8)	0.201
Digestive system	166 (15.9)	6 (4.8)	160 (16.5)	< 0.001
Immune system	35 (3.2)	1 (0.8)	34 (3.5)	0.176
Unknown	41 (3.8)	14 (11.2)	27 (2.8)	< 0.001
*Days in hospital, median (IQR)*	10 (4; 22)	5 (1; 11)	11 (4; 23)	< 0.001
*Days in ICU, median (IQR)*	4 (1; 11)	2 (0; 6)	4 (1; 12)	< 0.001
*ICU mortality, n (%)*	876 (80.2)	125 (100)	751 (77.7)	< 0.001
*Hospital mortality, n (%)*	941 (86.2)	125 (100)	816 (84.4)	< 0.001
*EPS score by ICU, median (IQR)*	7 (6; 8)	7 (6; 8)	7 (6; 9)	0.039
*Patients with decision-making capacity, n (%)*	126 (11.5)	1 (0.8)	125 (12.9)	< 0.001
*With advance directives, n (%)*	270 (26.9)	11 (11.2)	259 (28.6)	< 0.001
*With legal representatives, n (%)*	440 (43.7)	34 (35.1)	406 (44.6)	0.091
*Information available about patients’ treatment wishes*^c^	1042	93	949	
Yes, *n* (%)	816 (78.3)	31 (33.3)	785 (82.7)	< 0.001
If yes, from patient, *n* (%)	143 (17.5)	1 (3.2)	142 (17.5)	0.058
If yes, from family, *n* (%)	766 (93.9)	29 (93.5)	737 (93.9)	< 0.001
If yes, from other	49 (6)	4 (12.9)	45 (5.7)	0.207
*If patient desires were known, were they followed? n = yes (%)*	628 (98)	20 (95.2)	608 (98.1)	0.907

*EPS* End-of-life practice score, *IQR* Interquartile range

^a^*P*-values not assessed because of the many unknowns

^b^Multiple diagnoses possible

^c^number of patients with available data

### Patients with treatment limitations

Among the study population, 967 (88.6%) patients had treatment limitations, including 556 (50.9%) patients with decisions to withdraw and 411 (37.6%) patients with decisions to withhold life-sustaining treatment. Among 125 patients without limitations, 33 (3.0%) patients suffered brain death and 92 (8.4%) died despite cardiopulmonary resuscitation (Table 2). Compared to patients without treatment limitations, patients with treatment limitations were older (median age: 73 years [IQR 61–80] vs. 68 years [IQR 54–77]; *p* < 0.001), more often had decision-making capacity (12.9% vs. 0.8%, *p* < 0.001), had more advance directives (28.6% vs.11.2%, *p* < 0.001) and were more frequently treated in ICUs with higher total EPS scores (*p* = 0.039). The patients with treatment limitations more often had pre-existing neurological and digestive system diseases. Reasons for ICU admission were more often respiratory (38.6%) and less often cardiovascular (19.4%). There was no difference in patient sex, diagnosis of cancer, and the presence of a legal representative. Patients with treatment limitations had longer ICU and hospital stays (4 vs. 2 days, *p* < 0.0001 and 11 vs. 5 days, *p* < 0.0001, respectively); 77.7% (751) died in the ICU and 84.4% (816) died in the hospital (Table 1). The mortality rate of patients with decision to withdraw treatment was higher 98.5% (547) than that of patients with decision to withhold treatment (65.5% [269], *p* < 0.001; Table 2).

**Table 2:** Patients by end-of-life categories

*N total* *=* *1092*	*n* available data	Brain death	CPR	WD	WH	*P*
Patients, (%)	1092	33 (3.0%)	92 (8.4%)	556 (50.9%)	411 (37.6%)	
Age, years, median (IQR)	1092	63 (47; 75)	68 (59; 77)	71 (59; 79)	75 (64; 82.5)	< 0.001
Male, *n* (%)	1092	19 (57.6)	57 (62)	340 (61.1)	231 (56.2)	0.139
Sepsis at admission, *n* (%)	1092	0	19 (21)	136 (24)	74 (18)	0.020
Advance directive, *n* (%)	1004	1 (3.3)	10 (14.7)	146 (28.3)	113 (29)	0.881
Time between hospital admission and first treatment limitation, median (IQR)	125	–	–	8 days 0 h (1 day 21 h; 19 days 5 h)	4 days 10 h (0 day 21 h; 13 days 21 h)	< 0.001
Time between ICU admission and first treatment limitation, median (IQR)	125	–	–	3 days 2 h (0 day 15 h; 10 days 22 h)	1 day 0 h (0 day 2 h; 4 days 20 h)	< 0.001
Time between first treatment limitation and death, days, median (IQR)	276	–	–	0 day 15 h (0 day 3 h; 2 days 5 h)	0 day 23 h (0 day 3 h; 3 days 12 h)	0.029
Days in hospital, median (IQR)	–	2 (1; 8)	6 (2; 11)	11 (4; 23)	12 (5; 23)	0.083
Days in ICU, median (IQR)	–	1 (0; 4)	2 (0; 6.2)	5 (2; 14)	4 (1; 10)	0.009
ICU mortality, *n* (%)	–	–	–	523 (93.9%)	218 (53.0%)	< 0.001
Hospital mortality, *n* (%)	–	33 (100)	92 (100)	547 (98.5)	269 (65.5)	< 0.001

*p*-Values were calculated for the comparison of patients with WD and with only WH decisions

*CPR* cardiopulmonary resuscitation, *WD and WH* withdrawal and withholding of life-sustaining therapy, respectively, *IQR* interquartile range, *ICU* Intensive care unit

**Fig. 2:**
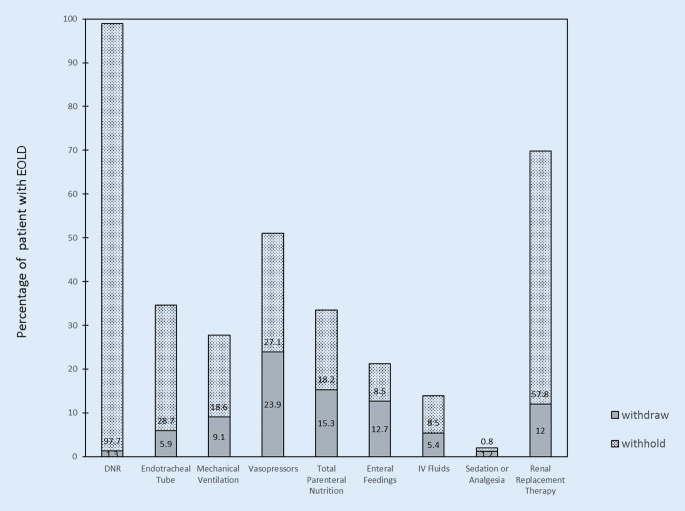
Treatment limitations at the time of first decision to withhold or withdraw life-supporting therapy. *DNR* Do not resuscitate; *IV* intravenous, *DNR Withhold* means to withhold cardiopulmononary resuscitation. *DNR Withdraw* means that the order for DNR is removed

Treatment limitations at the time of the first decision to withhold or withdraw life-supporting therapy are shown in Fig. 2. The most common treatments that were withheld were CPR (855 [97.7%]), renal replacement therapy (328 [57.8%]), endotracheal intubation (241 [28.7%]), or vasopressors (214 [27.1%]). The most common treatments that were withdrawn were vasopressors (189 [23.9%]), total parenteral nutrition (47 [15.3%]), enteral feedings (73 [12.7%]), renal replacement therapy (68 [12%]), and mechanical ventilation (78 [9.1%]).

The proportion of advance directives was not different between patients with withdrawing or withholding treatments (Table 2).

### Practice of decision-making

Information about presumed treatment desires was available in 816 patients (78.3%) and more often for patients with treatment limitations than those without (785 [82.7%] vs 31 [33.3%], *p* < 0.0001; Table 1). If patient desires were known, they were usually followed (628 [98%]), but they were actually known in only 641 (58.7%) of patients, suggesting uncertainty about the patient wishes in the remaining 451 patients (41.3%; Table 1). In patients with treatment limitations, physicians obtained information mostly from the families (766 [93.9%]) and only in 143 cases (17.5%) from the patients themselves.

Treatment limitation discussions were mostly initiated by ICU physicians (777 patients [80.4%]). Nurses rarely brought up the topic (1 [0.1%]). Physicians discussed most treatment limitations with patients capable of mental decision-making (115 [91.3%]) and families (798 [82.6%]), and in doing so commonly applied shared decision-making (104 [90.4%] and 603 [75.8%], respectively). Physicians discussed 88.1% (852/967) of treatment limitations with other ICU physicians and 66.3% (641/967) with nurses. Agreement was high between all stakeholders and among family members, and a delay in decision-making due to disagreement was rare (37 [4.2%]; Table 3).

**Table 3:** Practice of decision making for patients with treatment limitations (*n* = 967)

	All patients with available data	*n* (%)
*Who first brought up the topic?*		
ICU physicians	967	777 (80.4)
Primary physicians	967	47 (4.9)
Nurses	967	1 (0.1)
Consulting physicians	967	48 (5.0)
Patients	967	39 (4.0)
Families	967	55 (5.7)
*Discussion of treatment limitations*		
With the patient	126	115 (91.3)
- If yes, … was patient told?	115	3 (2.6)
… was patient asked?	115	8 (6.9)
… was there shared decision-making?	115	104 (90.4)
With the family	967	798 (82.6)
- If yes, … was family told?	798	149 (18.7)
… was family asked?	798	44 (5.5)
… was there shared decision-making?	798	603 (75.8)
- If no, because … family won’t understand	168	7 (4.2)
… unavailable	168	32 (19)
… no family	168	41 (24.4)
… patient unresponsive to maximal therapy	168	63 (37.5)
With others		
- ICU physicians	967	852 (88.1)
- Primary physicians	967	247 (25.2)
- Consulting physicians	967	327 (33.8)
Nurses	967	641 (66.3)
*Agreement was present …*		
Between physicians and nurses	826	823 (99.6)
Between clinicians and family	746	733 (98.3)
Among family members	679	675 (99.4)
Between clinicians and patient	176	165 (93.8)
Between ICU physicians and other physicians	800	787 (98.4)
*Delay in decision-making because of disagreement*	873	37 (4.2)
Written order for DNR decision	963	858 (89.1)
Documentation of DNR or NoCPR in medical record	963	934 (97)

*DNR* do not resuscitate, *CPR* cardiopulmonary resuscitation, *ICU* intensive care unit

Supplemental table 3 shows the reasons, considerations, and difficulties of end-of-life decision-making. Physicians responsible for decision-making stated that the primary reason for limiting treatment was unresponsiveness to maximal therapy (335 [34.6%]). Patient or family requests were named in 140 (14.5%) and 27 (2.8%), respectively. Poor quality of life was the primary reason in 39 (4%) patients, while age was rarely the primary reason (4 [0.4%]). Primary considerations for decision-making were mostly based on the ethical principles of good medical practice (505 [52.3%]), best interest of the patient [25] (258 [26.7%]), or autonomous patient decision/advance directive (180 [18.6%]). Economic, religious, social, or legal concerns were not mentioned. Almost all physicians (945 [97.7%]) reported that they had no difficulty about either withholding or withdrawing treatment.

### Time intervals

The median time interval between ICU admission and first treatment limitation was 2 [IQR 0–8] days, and between the first treatment limitation and death 1 [IQR 0–3] day. The time between ICU admission and first end-of-life decision was significantly shorter for patients with advance directives (median: 1 day 21 h [0 day 5 h to 5 days 21 h]) compared to patients without advance directives (median: 2 days 13 h [0 day 10 h to 9 days 23 h]; *p* < 0.05). In contrast, the time between first treatment limitation and death was significantly longer for patients with advance directives (median: 1 day 3 h [0 day 6 h to 3 days 1 h]) than for patients without advance directives (median: 0 day 16 h [0 day 2 h to 2 day 6 h]; *p* < 0.001). Decisions to withhold treatments (1 day [0 h to 4 days 20 h]) in the ICU were made in median 2 days earlier than decisions to withdraw treatments (3 days 2 h [15 h to 10 days 22 h]; *p* < 0.001; Table 2).

## Discussion

The results of this study showed that among 1092 patients in German ICUs who died or had limitations of life-sustaining therapy, decisions to limit life support preceded 9 out of 10 deaths, and only 8% of deaths occurred after full cardiopulmonary resuscitation (failed CPR). It is noteworthy that 22% of patients with a limitation of life-sustaining therapy were discharged alive from the ICU.

Treatment limitations occurred more often in older patients, in patients with advance directives, or decision-making capacity. Physicians commonly sought information about patient wishes, based their decision-making on shared decision-making with patient and families and ethical considerations. They reported having no difficulties with either withholding or withdrawing life support. Limitations also occurred more often in ICUs with a higher Ethical Practice score, suggesting the importance of palliative structures like local ethical standards and written practice guidelines to improve decision-making confidence for practitioners in the palliative situation.

Our findings illustrate the growing importance of palliative care in German ICUs. It has become an everyday occurrence, but there is lack of recognition of its importance. Out data point to some opportunities for improvement. Treatment limitations in German ICUs occur more frequently and failed CPR less frequently than before. Previously, a retrospective German study from 2002–2006 found that only 29% of deaths were preceded by end-of-life decisions and only 3.5% of patients survived end-of-life decisions [18]. This is in contrast to 88.6% of decisions to withdraw and withhold and a hospital survival rate of 13.8% in the study we presented here. Compared to worldwide data from Ethicus‑2, failed CPR occurred in a similar range in North American ICUs (8.5%) but less often than in ICUs in Australia/New Zealand (4.3%) or Northern Europe (3.7%) [3].

Palliative care in the ICU is increasingly provided through interdisciplinary team meetings, integration of palliative care specialists, ethics consultation and family conferences [22]. Ethical principles and practices of palliative care in the ICU have been outlined by national medical societies [20]. However, although most German intensivists practice palliative care, only a minority feels confident doing so [2]. This may be due to a perceived lack of structures and standards which support the change from curative to palliative care, namely lack of interdisciplinary or ethics case reviews, palliative care training or standard operating procedures for end-of-life care. Indeed, the EPS (end-of-life practice score) which assesses end-of-life protocols and palliative care consultations seems to suggest that treatment limitations occurred more often in ICUs with a higher EPS. However, this association needs to be treated with caution since more research is needed to understand the validity of this novel score.

Our data suggest a perceived gap between available information about patient wishes and unambiguous directives for decision-making. Physicians had information about patient wishes and discussed treatment limitations with families in over 80% of patients. They perceived that the patient will was followed in about 60% of patients. However, the question about the patient desires remained unanswered in about 40%, leading to the assumption that the patient’s will remained unclear despite discussion with families. In our study, 27% of patients had advance directives, which are legally binding in Germany. This is similar to recent findings from the University of Hamburg [8] but lower than the prevalence in North America (49%) reported in the worldwide Ethicus‑2 study [9]. However, the prevalence of advance directives may not be high enough to support patient-oriented end-of-life decisions in most patients. Furthermore, the advance directives that are in use in Germany often contain unspecific wording which makes them unsuitable for many acute situations [15]. More and better advance directives are urgently needed in an ageing society. We speculate that the patient’s will remained unclear in a considerable proportion of ICU patients. Given Germany’s aging population and the increasing proportion of elderly patients who receive intensive care treatment at the end-of-life [10], avoiding inappropriate intensive care is a growing challenge.

If physicians did not speak with the family, this was often because the family was not available. Data also suggest that ICU physicians involve most patients and families in the decision-making. However, the most common primary reasons for treatment limitations were unresponsiveness to maximal therapy, severity of disease, or underlying comorbidity. This suggests a more physician-centered approach with the intent to avoid nonbeneficial treatments regarding ethical principles such as good medical practice or best interest of the patient as primary consideration. This discrepancy can be explained with the uncertainty of patient wishes and family needs. According to a recent survey, 11% of families felt overwhelmed and wanted less participation in decision-making [12]. It would be desirable to have regular meetings between treating physicians and nurses, patients and relatives and other doctors engaged in the patient’s care like family doctors, so that the patient’s wishes can be evaluated according to the disease course and achievable therapeutic options.

Our study cannot answer whether end-of-life decisions were timely or delayed. Physicians declined a delay due to disagreements between health care providers. The first treatment limitation occurred 2 days after ICU admission and death occurred 1 day after the first limitations. These intervals are comparable to findings from other studies [26, 29].

End-of-life decision-making is considered a team effort, but in our study most decisions were reported to be initiated by physicians—except in one case, nurses were never reported to bring up the topic first. Nurse initiation was even lower than in the 1999–2000 (Ethicus-1) study [6] and declined across all European ICUs [5]. On the other hand, two-thirds of decisions were discussed with nurses and nearly total agreement with decisions was reported.

Physicians in the present study reported not having difficulties with withholding or withdrawing therapy. This is notable because withholding therapy is sometimes considered to be psychologically easier and more passive than withdrawing treatment [17]. However, a recent prospective multicenter study in 43 French ICUs in 2013 showed a similar pattern [16]. Given that only a decade ago in Germany, the issue of limiting life support was discussed in a controversial manner with concerns that limiting life support could be illegal [4], this finding indicates that the practice of limiting nonbeneficial treatment has become more accepted and reflective of national recommendations [13]. The answers were given by senior physicians in the present study. Thus, this answer should not be transferred on younger colleagues, who should not be left alone with these decisions unless adequately trained.

In France, Quenot et al. found similar physicians’ perceptions of nonbeneficial therapy, including exhaustion of therapeutic options and terminal status of chronic disease. In French ICUs, physicians also addressed age as a factor which determines nonbeneficence [21], whereas physicians in our study rarely reported age to be the primary reason for an end-of-life decision. Physicians mostly stated ethical principles such as good medical practice or best interest of the patient as primary consideration in decision making, and not economic or social obligations.

We can only speculate on the surprisingly high ICU and hospital survival rate (22% and 16%, respectively) after treatment limitation. A similar phenomenon in the Ethicus‑2 comparison study in European ICUs was discussed as result of decisions made before or during hospitalization due to the patients’ wishes [24]. A study in Finnish ICUs found that one in four patients survived 1 year, depending on housing type, prehospital fitness, and the need of postoperative care in an ICU [1].

Our study has strengths and limitations. To our knowledge, this is the largest prospective and patient-based study of end-of-life decisions in the ICU in Germany. Data were collected centrally, submitted to quality controls, and used in previous and international studies [23, 24], thus, enabling comparison. The study also has limitations. Participating ICUs were predominantly academic and self-selected on account of their ethical interest which introduces selection bias. Thus, findings may not be generalizable. Moreover, the collected data did not elicit the perceptions of nurses. This may have introduced bias since nurses perceive end-of-life decision-making more negatively than physicians [14]. Answers were given by senior physicians; thus, they may not reflect the uncertainty experienced by younger physicians. Moreover, self-reported answers to ethical questions may underlie social desirability bias. We left the classification of whether decision discussions were shared to the responsible physician. Thus, we cannot rule out misclassification bias. Finally, our findings cannot be extrapolated to other countries where there is less limitation of life-supporting therapies due to different cultures, healthcare systems, and population demographics.

## Conclusions

In German ICUs, decisions to limit life support precede nine out of 10 deaths, and 22% of patients with a limitation of life-sustaining therapy survive the ICU. Physicians often seek information about patient wishes, base their decision-making on discussions about prognosis and ethical considerations, and have no difficulties with either withholding or withdrawing life support. However, our findings suggest that treatment preferences of nearly half of the patients remain unknown and fail to guide treatment decisions. Further work should investigate structured approaches to implement palliative care, validate the ethical practice score, and explore timing and nature of discussions. More efforts are needed to increase the appropriateness and prevalence of advance directives.

### Supplementary Information

Supplementary Tables 1–3

## Appendix

### Participating centers and investigators

- Augsburg: Klinik für Anästhesiologie und Operative Intensivmedizin, Klinikum Augsburg, Dr. med. Ulrich Jaschinski, Ilse Kummer
- Berlin: Bundeswehrkrankenhaus Berlin, Klinik für Anästhesie, Intensivmedizin, Notfallmedizin und Rettungsdienst, Oberstabsarzt Katharina Buchholzer, Oberfeldarzt Karin Dey
- Berlin: Charité Universitätsmedizin Berlin, Klinik für Anästhesiologie mit Schwerpunkt operative Intensivmedizin Prof. Dr. med. Claudia Spies, Chirurgische Intensivstation Beatrix Laetsch, Dr. Katrin Schmidt
- Berlin: Charité Universitätsmedizin Berlin, Klinik für Anästhesiologie mit Schwerpunkt operative Intensivmedizin Prof. Dr. med. Claudia Spies, neurochirurgische Intensivstation Elke Falk, Susanne Sonnema, Dr. Susanne Joebges
- Dresden: Klinik und Poliklinik für Anästhesiologie und Intensivtherapie, Universitätsklinikum Carl Gustav Carus Dresden, Prof. Dr. Max Ragaller
- Jena: Klinik für Anästhesiologie und Intensivmedizin, Universitätsklinik Jena, PD Dr. med. Christiane Hartog, Dipl. Psych. Daniel Schwarzkopf, Franziska Hoffmann, Dipl.-Psych. Anna Mikolajetz, Swastika Kharel, Steffi Kolanos
- Leipzig: Klinik und Poliklinik für Anästhesiologie und Intensivtherapie, Universitätsklinikum Leipzig, PD Dr. med. habil. Sven Bercker, Dr. med. Philipp Simon
- München: Klinik für Anästhesiologie, Klinikum der Ludwig-Maximilians-Universität (LMU) München, Prof. Dr. med. Josef Briegel, PD Dr. med. Volker Huge, Gunnar Rehse
- Ulm: Klinik für Anästhesiologie und Intensivmedizin, Universitätsklinikum Ulm, Prof. Dr. med. Manfred Weiss, Prof. Dr. med. Eberhard Barth, Soelvi Barth
- Tettnang: Klinik für Anästhesiologie, Intensivmedizin, Notfallmedizin und Schmerztherapie, Medizincampus Bodensee—Klinik Tettnang, Dr. Andrej Michalsen
- Tübingen: Medizinische Klinik, Universitätsklinikum Tübingen, Prof. Dr. med. Reimer Riessen, Anna Heigl

## Declarations

For this article no studies with human participants or animals were performed by any of the authors. All studies mentioned were in accordance with the ethical standards indicated in each case.
