# Supplementary material for: End-of-life practices in 11 German intensive care units: Results from the ETHICUS-2 study
Source: Med Klin Intensivmed Notfmed. 2022 Sep 28;118(8):663–73. doi: 10.1007/s00063-022-00961-1 (PMC10624715; doi:10.1007/s00063-022-00961-1)
Supplement: Supplementary file 1 — Supplementary Tables 1–3 [file 63_2022_961_MOESM1_ESM.docx]

**Online Supplement**

**End-of-life Practices in German Intensive Care Units – Results from the ETHICUS-2 Study**

C. Denke (1), U. Jaschinski (2); R. Riessen (3); S. Bercker (4); C. Spies (1); M. Ragaller (5); M. Weiss (6); K. Dey (7); A. Michalsen (8); J. Briegel (9); A. Pohrt (10); C. L. Sprung (11); A. Avidan (11); C. S. Hartog (1)(12) for the SepNet Critical Care Trials Group - Ethicus II study group.

**Supplementary Table 1**

End-of-life categories were mutually exclusive and defined as follows:

| Withholding treatment (WH) | a decision not to start or to escalate a life- sustaining therapy |
| --- | --- |
| Withdrawing treatment (WD) | a decision to stop a life-sustaining therapy presently being given |
| Shortening the dying process (SDP) | acting with specific intent of shortening the dying process. |
| Failed Cardiopulmonary resuscitation (failed CPR) | death despite full cardiac support and ventilation |
| Brain death (BD) | documented cessation of cerebral function and meeting criteria for brain death |

**Supplementary Table 2**

Ethical Practice Score

| (1) routine family meetings; |
| --- |
| (2) daily deliberation for the appropriate level of care; |
| (3) end-of-life discussions during meetings; |
| (4) written triggers for limitations; |
| (5) written end-of-life guidelines; |
| (6) written protocols; |
| (7) palliative care consultations; |
| (8) ethics consultations; |
| (9) staff taking communications courses; |
| (10) staff taking bioethics courses; |
| (11) national end-of-life guidelines; |
| (12) national legislation. |

**Supplementary Table 3**

End-of-life decision-making: reasons, considerations and difficulties

|  | Patients with therapy limitations  n = 967 |
| --- | --- |
| **Primary reason for withholding or withdrawing life-sustaining treatment N (%)** |  |
| Unresponsive to maximal therapy | 335 (34.6) |
| Neurologic | 171 (17.7) |
| Patient request | 140 (14.5) |
| Multi-organ failure | 101 (10.4) |
| Chronic disease | 94 (9.7) |
| Poor quality of life | 39 (4) |
| Family request | 27 (2.8) |
| Sepsis/septic shock | 19 (2) |
| Age | 4 (0.4) |
| **Primary consideration in decision-making** |  |
| Good medical practice* | 505 (52.3) |
| Best interest of the patient** | 258 (26.7) |
| Autonomous patient decision | 99 (10.2) |
| Advance directive | 81 (8.4) |
| Cost effectiveness | - |
| Need for an ICU bed | 1 (0.1) |
| Religious principles | - |
| Social pressures | - |
| Legal concerns | - |
| **Primary difficulty for physician in either withholding or withdrawing life-sustaining treatment** |  |
| Ethical | 2 (0.2) |
| Legal | 3 (0.3) |
| Disagreements | 11 (1.1) |
| Religious | 1 (0.1) |
| None | 945 (97.7) |

Explanations:

* Good medical practice: considering that maximal therapy is not beneficial for the patient; **Acting in the best interest of the patient: decision-making when the patient lacks capacity and the patient’s values and preferences are unknown
